# D-Dimer and Prothrombin Time Are the Significant Indicators of Severe COVID-19 and Poor Prognosis

**DOI:** 10.1155/2020/6159720

**Published:** 2020-06-16

**Authors:** Hui Long, Lan Nie, Xiaochen Xiang, Huan Li, Xiaoli Zhang, Xiaozhi Fu, Hongwei Ren, Wanxin Liu, Qiang Wang, Qingming Wu

**Affiliations:** ^1^Internal Medicine of Tianyou Hospital, Wuhan University of Science and Technology, Wuhan 430064, China; ^2^Institute of Infection, Immunology and Tumor Microenvironment, Hubei Province Key Laboratory of Occupational Hazard Identification and Control, Medical College, Wuhan University of Science and Technology, Wuhan 430065, China

## Abstract

**Objective:**

To investigate the value of coagulation indicators D-dimer (DD), prothrombin time (PT), activated partial thromboplastin time (APTT), thrombin time (TT), and fibrinogen (Fg) in predicting the severity and prognosis of COVID-19.

**Methods:**

A total of 115 patients with confirmed COVID-19, who were admitted to Tianyou Hospital of Wuhan University of Science and Technology between January 18, 2020, and March 5, 2020, were included. The dynamic changes of DD, PT, APTT, and Fg were tested, and the correlation with CT imaging, clinical classifications, and prognosis was studied.

**Results:**

Coagulation disorder occurred at the early stage of COVID-19 infection, with 50 (43.5%) patients having DD increased and 74 (64.3%) patients having Fg increased. The levels of DD and Fg were correlated with clinical classification. Among 23 patients who deceased, 18 had DD increased at the first lab test, 22 had DD increased at the second and third lab tests, and 18 had prolonged PT at the third test. The results from ROC analyses for mortality risk showed that the AUCs of DD were 0.742, 0.818, and 0.851 in three times of test, respectively; PT was 0.643, 0.824, and 0.937. In addition, with the progression of the disease, the change of CT imaging was closely related to the increase of the DD value (*P* < 0.01).

**Conclusions:**

Coagulation dysfunction is more likely to occur in severe and critically ill patients. DD and PT could be used as the significant indicators in predicting the mortality of COVID-19.

## 1. Introduction

COVID-19 which emerged in Wuhan, Hubei Province, China, is caused by severe acute respiratory syndrome coronavirus 2 (SARS-CoV-2). It is typically spread via respiratory droplets and during close contact. The main clinical manifestation is lung injury[1, 2]. Most of the patients have a favorable prognosis, but some rapidly progress to severe and critical cases with respiratory distress syndrome, coagulation dysfunction, multiple organ failure, etc.[3, 4]. Therefore, early identification of the severity is very important to the clinical diagnosis of and treatment for COVID-19. Commonly used clinical laboratory coagulation indexes, such as D-dimer (DD), prothrombin time (PT), activated partial thromboplastin time (APTT), thrombin time (TT), and fibrinogen (Fg), could sensitively reflect the clotting state of the body.

The aim of the report is to investigate role of the dynamic changes of DD, PT, APTT, TT, and Fg in predicting the severity and prognosis in patients with COVID-19.

## 2. Materials and Methods

### 2.1. Source of Patients and Diagnosis Criteria

The information of a total of 115 patients with confirmed COVID-19 who were admitted to Tianyou Hospital affiliated to the Wuhan University of Science and Technology between January 18, 2020, and March 5, 2020, was collected. The confirmed patients had a positive result of the nucleic acid test of SARS-CoV-2 by real-time fluorescence RT-PCR. Three clinical disease assessments were conducted using laboratory data collected. Cases of hospital discharge, death, and under treatment with a duration of hospitalization longer than 14 days prior to March 5, 2020, were studied. Cases with incomplete laboratory data or with a duration of hospitalization shorter than 14 days prior to March 5, 2020, were excluded. This study was approved by the Medical Ethics Review Board of Wuhan University of Science and Technology (No. 202009).

### 2.2. Clinical Classifications

#### 2.2.1. Case Identification

According to the Guidance for Corona Virus Disease 2019: Prevention, Control, Diagnosis, and Management edited by the National Health Commission of the People's Republic of China, all cases were identified into four categories of mild cases, ordinary cases, severe cases, and critical cases. (1) Mild cases had mild clinical symptoms and no pneumonia manifestation in imaging. (2) Ordinary cases had symptoms like fever and respiratory tract symptoms, and pneumonia manifestation can be seen in imaging. (3) Severe cases met any of the following: respiratory distress, RR ≥ 30 breaths/min; the oxygen saturation is less than 93% at a rest state; or arterial partial pressure of oxygen (PaO_2_)/oxygen concentration (FiO_2_) ≤ 300 mmHg (1 mmHg = 0.133 kPa). Patients with >50% lesion progression within 24 to 48 hours in pulmonary imaging were treated as severe cases. (4) Critical cases met any of the following: respiratory failure occurs, and mechanical ventilation is required; shock occurs; or complicated with other organ failure that requires monitoring and treatment in ICU.

#### 2.2.2. Outcome of Illness

According to clinical progression, outcomes in endpoints were divided into four types: hospital discharge, improved, exacerbation, and death.

### 2.3. Data Collection

The laboratory data were collected at three time points: admission, 3-5 days of hospitalization, and at the composite endpoint. DD, PT, APTT, and Fg were obtained and labeled as DD1-3, PT1-3, APTT1-3, TT1-3, and Fg1-3, respectively. Meanwhile, case identification, imaging identification, and outcome of illness were defined.

### 2.4. Statistical Methods

Statistical analysis was conducted using the SPSS 25.0 software. Descriptive statistics included means and standard deviations. The Kruskal-Wallis *H*-test and independent sample chi-square test were used to analyze differences between groups. The Receiver Operating Characteristic curve (ROC curve) was used to calculate the area under the curve (AUC) of DD and PT in order to evaluate the sensitivity and specificity of these factors in predicting mortality and hospital discharge. Spearman's rank correlation analysis was utilized to measure the degree of correlation between the hierarchically ordered variables in this study. A *P* value < 0.05 was considered statistically significant.

### 2.5. Patient and Public Involvement

This was a retrospective case series study, and no patients were involved in the study design, setting the research questions, or the outcome measures directly. No patients were asked to advise on the interpretation or writing up of results.

## 3. Results and Discussion

### 3.1. Demographic Characteristics

Among 115 patients with COVID-19, the median ages were 63.55 ± 13.86 (27-96) years old, male were 66 (57.4%) cases, female were 49 (42.6%) cases, and over 60 years old were 78 (67.8%) cases. At the time of admission, mild and ordinary patients were 39 (33.9%) cases, severe patients were 48 (41.7%) cases, and critical patients were 28 (24.3%) cases (Table 1). In this study, more patients were male and more patients were more than 60 years, consistently with previous literature report [1].

### 3.2. The Relationship between the Levels of DD1, PT1, APTT1, Fg1, and Clinical Classification

There are significant differences in DD1 between different clinical classifications (*P* < 0.05). The severity of the disease increased as DD1 increased. 81 (70.4%) patients had Fg1 increased (Table 2).

### 3.3. Relationship between the Dynamics Changes of DD, PT, APTT, TT, Fg, and the Prognosis of COVID-19

Significant difference (*P* < 0.05) and positive correlation were found between DD, PT, and outcomes at composite endpoints. Correlation in third detection was stronger than that in first and second detection.

Among 23 patients who died, 18 (78.3%) cases had DD1 increased, 12 of 18 had DD1 two times higher (>1.10 mg/L), 22 cases had DD2 and DD3 increased, 21 of 22 had DD2 and DD3 two times higher (>1.10 mg/L). Eight cases in exacerbated patients occurred increased DD2 and DD3 all higher (1.10 mg/L) (Table 3).

### 3.4. Analysis of DD and PT in Predicting Hospital Discharge and Mortality of COVID-19

**Figure 1:**
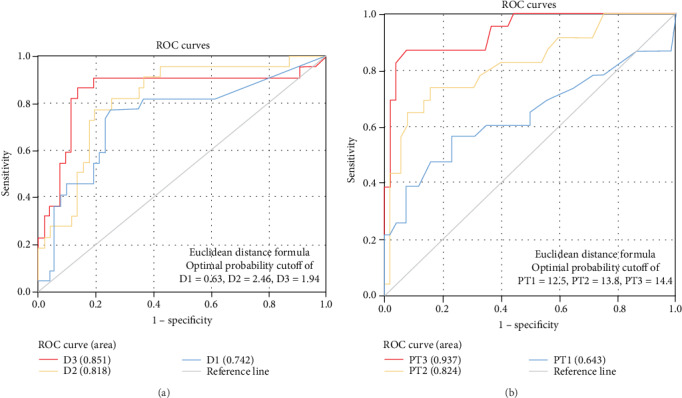
The relationship between DD, PT, and death. (a) ROC curve of DD1, DD2, and DD3 in predicting hospital discharge and mortality. (b) ROC curve of PT1, PT2, and PT3 in predicting hospital discharge and mortality.

We used the ROC curve analysis to evaluate the diagnostic value of hospital discharge and mortality in 115 patients. The AUCs of DD1, DD2, and DD3 to predict hospital discharge and mortality were 0.742, 0.818, and 0.851, respectively (Figure 1(a)). The AUCs of PT1, PT2, and PT3 to predict hospital discharge and mortality were 0.643, 0.824, and 0.937, respectively (Figure 1(b)).

### 3.5. Dynamic Changes of Chest CT Imaging, DD and CTA in COVID-19 Patients

At the early stage of the disease, the correlation between CT imaging changes and DD value was not obvious; however, with the progression of the disease, the change of CT was closely related to the increase of DD value, and there was a significant statistical difference (Table 4).

**Figure 2:**
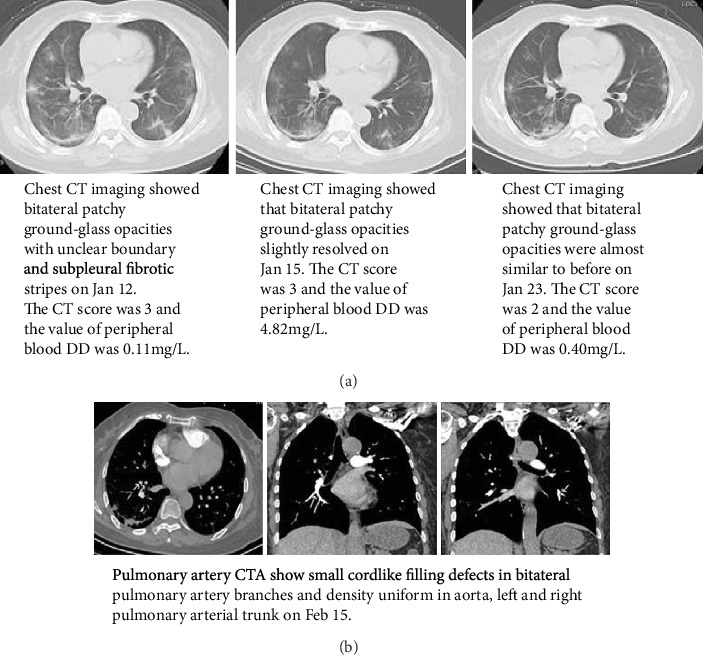
The changes of DD, CT, and COVID-19. (a) The dynamic changes of chest CT imaging and DD of patient Kang xx. (b) Pulmonary artery CTA of patient Kang xx.

The clinical observation showed that the abnormal coagulation factor was consistent with the CT imaging results. In this paper, a typical patient was taken as an example. The dynamic changes of chest CT imaging and DD were consistent (Figure 2(a)). Increased DD was associated with pulmonary embolism, which was confirmed by CTA (Figure 2(b)).

## 4. Conclusions

COVID-19 is an acute infectious disease caused by a new type of coronavirus (SARS-CoV-2). The onset of COVID-19 presents as fever, mild or sever, in a few cases [4–6]. Some patients may gradually develop dyspnea. However, in severe cases, the disease progresses rapidly, and patients develop severe septic shock and die [7–10]. The severity and prognosis of COVID-19 are complicated by the diversity of symptoms, radiological manifestations, and disease progression. It is particularly noteworthy that some severe, critical, and deceased patients have significant coagulation dysfunction [1, 4]. The pathological changes of the disease have been added into the seventh edition of the COVID-19 Treatment Plan issued by the National Health Commission of China, in which both autopsy and histopathologic examinations demonstrate thrombus or microthrombus in the lung, heart, kidney, and/or liver.

Upon SARS-CoV-2 entering the body through the angiotensin-converting enzyme 2 (ACE2) receptor adsorbed on the surface of mucosal epithelial cells [7, 8], its pathogen-associated molecular pattern (PAMP) can be quickly recognized by the immune system, and immune response is activated to clear the virus. However, overactivated immune response could cause a cytokine storm. As a result, cytokine storm causes vascular endothelial damage, activates the coagulation system, and inhibits the fibrinolytic and anticoagulating systems. Excessive thromboses in the microvascular system lead to disseminated intravascular coagulation (DIC) and, ultimately, microcirculatory disorder and serious multiple organ dysfunction syndrome [11]. Therefore, early detection and correction of coagulation dysfunction could effectively reduce mortality.

Commonly used laboratory coagulation indicators include DD, PT, APTT, and Fg. DD is the product of fibrinolytic solubilization of fibrin, and the elevated level of DD indicates that there is a hypercoagulating state and secondary fibrinolysis in the body, which can be seen in increased fibrinolytic activity of the body system [12–15]. PT and APTT are exogenous and endogenous coagulating system factors, which can be used for early diagnosis of DIC. Fg is a protein with coagulation function synthesized by the liver, which is an important substance in the process of coagulation and thrombosis. High level of Fg is an important indicator for a variety of thrombotic diseases. DD, PT, APTT, and Fg can be used as sensitive indicators to reflect different degrees of coagulating dysfunction. Therefore, in this article, the study was focused on if these indicators are related to the severity of COVID-19.

The results of this study showed that DD and Fg could be used as new indicators for the clinical classification of COVID-19. In the first test of DD, 50 of 115 patients had abnormal levels of DD (>0.55 mg/L), accounting for 43.5% (50/115). Of the 28 critically ill patients, 17 were >0.55 mg/L, accounting for 60.7%. (17/25), and 14 cases had two times more than the normal reference value. 70.4% (81/115) of the COVID-19 patients had abnormal concentration of Fg. Additionally, it is noticed that the level of Fg was significantly increased in severe and critically ill patients, with 70.3% of severe and critical patients (52/74) >4.00 g/L. The results of the study indicate that the levels of DD and Fg significantly increased in severe and critically ill patients, and some patients deteriorated during treatment, suggesting that COVID-19 patients, especially severe patients, have a high risk of thrombosis, which is consistent with previous reports [1, 4].

In addition, the results of this study also show a significant correlation between coagulating factors and disease outcome, suggesting DD, PT, and APTT could serve as diagnostic indicators for disease progression. Among the 23 patients who deceased, 18 had abnormal DD in the first test, accounting for 78.3% (18/23), among which 12 had DD level two times more than the normal reference value. In the second and third tests, 8 exacerbating cases had DD level > 1.10 mg/L. Additionally, among 23 deceased patients, 21 cases had DD level two times more than the normal reference value. In the first test of PT, there were two abnormalities (15 sec) in 8 aggravating patients whereas 5 abnormalities (15 sec) in 23 deceased patients. While in the second and third PT tests, there were 10 and 18 abnormalities (> 15 sec), respectively, in 23 deceased patients. The gradually increasing DD and PT levels suggest the significant correlation with disease progression.

Using discharged and deceased cases as the basis of positive division, the ROC curve analyses showed the areas under the curve (AUCs) were 0.742, 0.818, and 0.851, respectively. The third time of PT and APTT test had AUCs at 0.937 and 0.856, respectively, indicating that PT and APTT had great value in disease prognosis.

Based on the study results, the levels of D-dimer, PT, and APTT were significantly higher, whereas Fg in deceased cases was significantly lower than those in survival cases, suggesting the dynamic coagulating process in patients with COVID-19 is likely the hypercoagulating state followed by the activation of fibrinolysis. In this study, PT and APTT prolonged in 23 deceased patients, and the prolongation was more significant in the second and third tests, indicating the patients were in the transition from the high coagulating state into fibrinolytic state due to the excessive consumption of coagulating factors. Additionally, the study results showed DD, one of the fibrinolytic degradation products, gradually increased throughout the disease, indicating that the patients were possibly in hyperfibrinolytic state, which is consistent with Chen et al.'s report [16].

CT imaging has been regarded as a valuable tool in diagnosis and prognosis of COVID-19. The study results showed that DD was correlated with CT imaging in predicting the progression of disease. Specifically, the increased level of DD suggests hypercoagulating state and the possible pulmonary embolism, which could be further confirmed by CT angiography (CTA).

One limitation of this study exists on that it was carried out in a single medical center with absence of the control group design due to the emergent situation of COVID-19 breakout. In the future, the researchers should integrate with a few medical centers in the area and draw the control group to boost the reliability of the study.

In conclusion, the results of this study showed that hypercoagulation was likely present in patients with COVID-19 at the early stage. And hypercoagulation is closely related to disease progression and clinical outcome. Therefore, the coagulation indicators such as DD and PT should be monitored as early as possible in order to detect thrombotic complications. It is imperative to take preventive treatment to reduce the risk of thromboembolism and DIC secondary to coagulation disorder, thereby decreasing the morbidity and mortality of COVID-19-infected patients.

## Figures and Tables

**Table 1:** Characteristics of patients with COVID-19.

Demographic	Clinical classifications on admission					Outcome at composite endpoint			
	Mild/ordinary cases	Severe cases	Critical cases	Total	Hospital discharge	Improved	Exacerbation	Death	Total
Age, years (*x* ± *s*)	57.08 ± 12.92	64.94 ± 12.75	70.18 ± 13.86	63.55 ± 13.86	59.42 ± 14.78	63.94 ± 12.24	67.75 ± 15.36	70.87 ± 10.00	63.55 ± 13.86
Distribution, *n* (%)									
<60	20 (17.4%)	11 (9.6%)	6 (5.2%)	37 (32.2%)	24 (20.9%)	8 (6.9%)	1 (0.8%)	4 (3.5%)	37 (32.2%)
≥60	19 (16.5%)	37 (32.2%)	22 (19.1%)	78 (67.8%)	28 (24.3%)	24 (20.9%)	7 (6.1%)	19 (16.5%)	78 (67.8%)
Total	39 (33.9%)	48 (41.7%)	28 (24.3%)	115	52 (45.2%)	32 (27.8%)	8 (6.9%)	23 (0.2%)	115
Gender									
Male, *n* (%)	20 (17.4%)	29 (25.2%)	17 (14.8%)	66 (57.4%)	28 (24.3%)	19 (16.5%)	7 (6.1%)	12 (10.4%)	66 (57.4%)
Female, *n* (%)	19 (16.5%)	19 (16.5%)	11 (9.6%)	49 (42.6%)	24 (20.9%)	13 (11.3%)	1 (0.8%)	11 (9.6%)	49 (42.6%)
Total	39 (33.9%)	48 (41.7%)	28 (24.3%)	115	52 (45.2%)	32 (27.8%)	8 (6.9%)	23 (0.2%)	115

**Table 2:** The first detection of DD1, PT1, APTT1, Fg1, and clinical classification.

Parameters	The first time clinical classifications (*n*, %)			Total
	Mild/ordinary cases	Severe cases	Critical cases	
DD1 (*M* ± SD)	0.85 ± 1.68	1.78 ± 4.40	3.86 ± 7.93	1.97 ± 5.01
<0.55	28 (24.3%)	26 (22.6%)	11 (9.6%)	65 (56.5%)
0.55-1.10	4 (3.4%)	8 (6.9%)	3 (2.6%)	15 (13.0%)
>1.10	7 (6.0%)	14 (12.2%)	14 (12.2%)	35 (30.5%)
Total	39 (33.9%)	48 (41.7%)	28 (24.3%)	115
*χ*^2^, *P*	*χ* ^2^ = 9.505	*P* < 0.05		
*r*, *P*	*r* = 0.268	*P* < 0.01		
PT1 (*M* ± SD)	12.34 ± 1.91	12.14 ± 1.16	13.70 ± 3.38	12.59 ± 2.21
<9.2	0 (0%)	0 (0%)	0 (0%)	0 (0%)
9.20-15.0	37 (32.1%)	47 (40.9%)	23 (20.0%)	107 (93.0%)
>15	2 (1.7%)	1 (0.8%)	5 (4.3%)	8 (7.0%)
Total	39 (33.9%)	48 (41.7%)	28 (24.3%)	115
*χ*^2^, *P*	*χ* ^2^ = 7.013	*P* < 0.05		
*r*, *P*	*r* = 0.162	*P* > 0.05		
APTT1 (*M* ± SD)	3.49 ± 9.17	36.47 ± 9.29	36.98 ± 8.60	35.59 ± 9.13
<21.00	1 (0.8%)	0 (0%)	1 (0.8%)	2 (1.7%)
21.00-37.00	27 (23.5%)	26 (22.6%)	13 (11.3%)	66 (57.4%)
>37.00	11 (9.6%)	22 (19.1%)	14 (12.2%)	47 (40.9%)
Total	39 (33.9%)	48 (41.7%)	28 (24.3%)	115
*χ*^2^, *P*	*χ* ^2^ = 5.545	*P* > 0.05		
*r*, *P*	*r* = 0.171	*P* > 0.05		
Fg1 (*M* ± SD)	4.38 ± 1.15	4.93 ± 1.26	4.40 ± 2.07	4.61 ± 1.48
<2.00	1 (0.8%)	0 (0%)	6 (5.2%)	7 (6.1%)
2.00-4.00	16 (13.9%)	13 (11.3%)	5 (4.3%)	34 (29.6%)
>4.00	22 (19.1%)	35 (30.4%)	17 (14.8%)	74 (64.3%)
Total	39 (33.9%)	48 (41.7%)	28 (24.3%)	115
*χ*^2^, *P*	*χ* ^2^ = 18.661	*P* < 0.01		
*r*, *P*	*r* = 0.006	*P* > 0.05		
TT1 (*M* ± SD)				
<10	0 (0%)	1 (0.8%)	0 (0%)	1 (0.8%)
10-20	37 (32.2%)	47 (40.9%)	26 (22.7%)	110 (95.8%)
>20	2 (1.7%)	0 (0%)	2 (1.7%)	4 (3.4%)
Total	39 (33.9%)	48 (41.7%)	28 (24.4%)	115
*χ*^2^, *P*	*χ* ^2^ = 4.503	*P* > 0.05		
*r*, *P*	*r* = 0.175	*P* > 0.05		

Normal reference values: DD (<0.55 mg/L); PT (9.20-15 sec); APTT (21.00-37.00 sec); TT (10-20 sec); Fg (2.00-4.00 g/L). ^∗^*P* value was calculated by a 2-sided test.

**Table 3:** Correlation between the dynamics changes of DD, PT, APTT, Fg, and the prognosis of COVID-19.

Parameters	Outcome at composite endpoint (*n*)				Total
	Hospital discharge	Improved	Exacerbation	Death	
DD1 (*M* ± SD)	0.87 ± 1.73	1.55 ± 3.93	6.51 ± 10.29	3.47 ± 7.41	1.97 ± 5.01
<0.55	38	18	4	5	65
0.55-1.11	3	6	0	6	15
>1.11	11	8	4	12	35
Total	52	32	8	23	115
*χ*^2^, *P*	*χ* ^2^ = 20.82	*P* < 0.01			
*r*, *P*	*r* = 0.346	*P* < 0.01			
DD2 (*M* ± SD)	1.62 ± 2.29	4.73 ± 8.02	12.40 ± 13.21	8.08 ± 10.96	4.50 ± 7.99
<0.55	20	5	0	1	26
0.55-1.11	12	11	0	1	24
>1.11	20	16	8	21	65
Total	52	32	8	23	115
*χ*^2^, *P*	*χ* ^2^ = 30.11	*P* < 0.01			
*r*, *P*	*r* = 0.439	*P* < 0.01			
DD3 (*M* ± SD)	1.27 ± 2.08	2.38 ± 4.27	6.22 ± 3.75	8.93 ± 10.91	3.40 ± 6.23
<0.55	26	11	0	2	39
0.55-1.10	11	9	0	0	20
>1.10	15	12	8	21	56
Total	52	32	8	23	115
*χ*^2^, *P*	*χ* ^2^ = 36.86	*P* < 0.01			
*r*, *P*	*r* = 0.467	*P* < 0.01			
PT1 (*M* ± SD)	11.91 ± 0.99	12.56 ± 1.84	13.41 ± 2.37	13.86 ± 3.68	12.59 ± 2.21
<9.2	0	0	0	0	0
9.20-15.0	52	31	6	18	107
>15	0	1	2	5	8
Total	52	32	8	23	115
*χ*^2^, *P*	*χ* ^2^ = 16.403	*P* < 0.01			
*r*, *P*	*r* = 0.331	*P* < 0.01			
PT2 (*M* ± SD)	12.97 ± 2.29	13.74 ± 4.28	14.23 ± 2.13	16.63 ± 5.06	14.00 ± 3.80
<9.2	0	0	0	0	0
9.20-15.0	50	28	5	13	96
>15	2	4	3	10	19
Total	52	32	8	23	115
*χ*^2^, *P*	*χ* ^2^ = 21.104	*P* < 0.01			
*r*, *P*	*r* = 0.399	*P* < 0.01			
PT3 (s)	12.72 ± 1.68	12.81 ± 2.45	16.56 ± 5.50	24.52 ± 15.20	15.37 ± 8.45
<9.2	0	0	0	0	0
9.20-15.0	50	30	5	5	90
>15	2	2	3	18	25
Total	52	32	8	23	115
*χ*^2^, *P*	*χ* ^2^ = 58.66	*P* < 0.01			
*r*, *P*	*r* = 0.595	*P* < 0.01			
APTT1 (*M* ± SD)	36.55 ± 8.75	34.95 ± 9.51	32.09 ± 5.27	35.53 ± 10.54	35.59 ± 9.13
<21.00	0	0	0	2	2
21.00-37.00	27	21	7	11	66
>37.00	25	11	1	10	47
Total	52	32	8	23	115
*χ*^2^, *P*	*χ* ^2^ = 12.884	*P* < 0.05			
*r*, *P*	*r* = −0.131	*P* > 0.05			
APTT2 (*M* ± SD)	28.56 ± 6.48	27.79 ± 4.93	27.66 ± 3.42	32.98 ± 8.53	29.17 ± 6.63
<21.00	1	1	0	0	2
21.00-37.00	48	29	8	19	104
>37.00	3	2	0	4	9
Total	52	32	8	23	115
*χ*^2^, *P*	*χ* ^2^ = 4.857	*P* > 0.05			
*r*, *P*	*r* = 0.122	*P* > 0.05			
APTT3 (*M* ± SD)	28.78 ± 4.18	27.07 ± 3.38	29.44 ± 4.92	40.40 ± 13.80	30.67 ± 8.58
<21.00	1	0	0	1	2
21.00-37.00	48	32	7	10	97
>37.00	3	0	1	12	16
Total	52	32	8	23	115
*χ*^2^, *P*	*χ* ^2^ = 38.632	*P* < 0.01			
*r*, *P*	*r* = 0.359	*P* < 0.01			
TT1					
<10	0	0	1	0	1
10-20	51	32	7	20	110
>20	1	0	0	3	4
Total	52	32	8	23	115
*χ*^2^, *P*	*χ* ^2^ = 21.510	*P* < 0.01			
*r*, *P*	*r* = 0.225	*P* < 0.05			
TT2					
<10	0	0	0	0	0
10-20	52	31	8	20	111
>20	0	1	0	3	4
Total	52	32	8	23	115
*χ*^2^, *P*	*χ* ^2^ = 8.442	*P* < 0.05			
*r*, *P*	*r* = 0.167	*P* > 0.05			
TT3					
<10	0	0	0	0	0
10-20	51	32	8	20	111
>20	1	0	0	3	4
Total	52	32	8	23	115
*χ*^2^, *P*	*χ* ^2^ = 8.084	*P* < 0.05			
*r*, *P*	*r* = 0.136	*P* > 0.05			
Fg1 (*M* ± SD)	4.49 ± 1.29	4.81 ± 1.31	5.30 ± 1.44	4.39 ± 2.00	4.61 ± 1.48
<2.00	1	2	0	4	7
2.00-4.00	19	8	1	6	34
>4.00	32	22	7	13	74
Total	52	32	8	23	115
*χ*^2^, *P*	*χ* ^2^ = 9.81	*P* > 0.05			
*r*, *P*	*r* = −0.02	*P* > 0.05			
Fg2 (*M* ± SD)	3.55 ± 1.31	3.86 ± 1.32	3.84 ± 1.45	3.24 ± 1.80	3.60 ± 1.43
<2.00	3	1	1	8	13
2.00-4.00	35	20	3	9	67
>4.00	14	11	4	6	35
Total	52	32	8	23	115
*χ*^2^, *P*	*χ* ^2^ = 18.92	*P* < 0.01			
*r*, *P*	*r* = −0.09	*P* > 0.05			
Fg3 (*M* ± SD)	3.11 ± 1.03	3.96 ± 1.42	4.13 ± 2.49	3.24 ± 1.44	3.43 ± 1.41
<2.00	3	1	2	3	9
2.00-4.00	42	19	3	15	79
>4.00	7	12	3	5	27
Total	52	32	8	23	115
*χ*^2^, *P*	*χ* ^2^ = 13.28	*P* < 0.05			
*r*, *P*	*r* = 0.07	*P* > 0.05			

Normal reference values: DD (<0.55 mg/L); PT (9.20-15 sec); APTT (21.00-37.00 sec); TT (10-20 sec); Fg (2.00-4.00 g/L). ^∗^*P* value was calculated by a 2-sided test.

**Table 4:** Correlation analysis between DD and chest CT in the same period.

The different stages of CT	DD			
	<0.55	0.55-1.10	>1.10	Total
CT1				
Normal	3	0	2	5
Mild	16	1	4	21
Progressive	29	10	16	55
Severe	17	4	13	34
Total	65	15	35	115
*χ*^2^, *P*	*χ* ^2^ = 6.514	*P* > 0.05		
*r*, *P*	*r* = 0.152	*P* > 0.05		
CT2				
Normal	1	0	0	1
Mild	6	7	4	17
Progressive	16	14	23	53
Severe	1	2	23	26
Total	24	23	50	97
*χ*^2^, *P*	*χ* ^2^ = 24.340	*P* < 0.01		
*r*, *P*	*r* = 0.498	*P* < 0.01		
CT3				
Normal	0	0	0	0
Mild	19	10	4	33
Progressive	10	4	13	27
Severe	1	2	5	8
Total	30	16	22	68
*χ*^2^, *P*	*χ* ^2^ = 13.501	*P* < 0.01		
*r*, *P*	*r* = 0.423	*P* < 0.01		

## Data Availability

The data used to support the findings of this study are available from the corresponding author upon request.
